# Scaling Properties of Dimensionality Reduction for Neural Populations and Network Models

**DOI:** 10.1371/journal.pcbi.1005141

**Published:** 2016-12-07

**Authors:** Ryan C. Williamson, Benjamin R. Cowley, Ashok Litwin-Kumar, Brent Doiron, Adam Kohn, Matthew A. Smith, Byron M. Yu

**Affiliations:** 1 Center for the Neural Basis of Cognition, Carnegie Mellon University, Pittsburgh, Pennsylvania, United States of America; 2 School of Medicine, University of Pittsburgh, Pittsburgh, Pennsylvania, United States of America; 3 Department of Machine Learning, Carnegie Mellon University, Pittsburgh, Pennsylvania, United States of America; 4 Center for Theoretical Neuroscience, Columbia University, New York City, New York, United States of America; 5 Department of Mathematics, University of Pittsburgh, Pittsburgh, Pennsylvania, United States of America; 6 Dominick Purpura Department of Neuroscience, Albert Einstein College of Medicine, Bronx, New York, United States of America; 7 Department of Ophthalmology and Vision Sciences, Albert Einstein College of Medicine, Bronx, New York, United States of America; 8 Department of Systems and Computational Biology, Albert Einstein College of Medicine, Bronx, New York, United States of America; 9 Department of Ophthalmology, University of Pittsburgh, Pittsburgh, Pennsylvania, United States of America; 10 Department of Bioengineering, University of Pittsburgh, Pittsburgh, Pennsylvania, United States of America; 11 Fox Center for Vision Restoration, University of Pittsburgh, Pittsburgh, Pennsylvania, United States of America; 12 Department of Electrical and Computer Engineering, Carnegie Mellon University, Pittsburgh, Pennsylvania, United States of America; 13 Department of Biomedical Engineering, Carnegie Mellon University, Pittsburgh, Pennsylvania, United States of America; The University of Texas at Austin, UNITED STATES

## Abstract

Recent studies have applied dimensionality reduction methods to understand how the multi-dimensional structure of neural population activity gives rise to brain function. It is unclear, however, how the results obtained from dimensionality reduction generalize to recordings with larger numbers of neurons and trials or how these results relate to the underlying network structure. We address these questions by applying factor analysis to recordings in the visual cortex of non-human primates and to spiking network models that self-generate irregular activity through a balance of excitation and inhibition. We compared the scaling trends of two key outputs of dimensionality reduction—shared dimensionality and percent shared variance—with neuron and trial count. We found that the scaling properties of networks with non-clustered and clustered connectivity differed, and that the *in vivo* recordings were more consistent with the clustered network. Furthermore, recordings from tens of neurons were sufficient to identify the dominant modes of shared variability that generalize to larger portions of the network. These findings can help guide the interpretation of dimensionality reduction outputs in regimes of limited neuron and trial sampling and help relate these outputs to the underlying network structure.

## Author Summary

We seek to understand how billions of neurons in the brain work together to give rise to everyday brain function. In most current experimental settings, we can only record from tens of neurons for a few hours at a time. A major question in systems neuroscience is whether our interpretation of how neurons interact would change if we monitor orders of magnitude more neurons and for substantially more time. In this study, we use realistic networks of model neurons, which allow us to analyze the activity from as many model neurons as we want for as long as we want. For these models, we found that we can identify the salient interactions among neurons and interpret their activity meaningfully within the range of neurons and recording time available in current experiments. Furthermore, we studied how the neural activity from the models reflects how the neurons are connected. These results help to guide the interpretation of analyses using populations of neurons in the context of the larger network to understand brain function.

## Introduction

Dimensionality reduction methods (for review, see [1]) have revealed compelling descriptions of neural mechanisms underlying decision-making [2, 3], motor control [4, 5], olfaction [6], working memory [7], visual attention [8], audition [9], rule learning [10], and speech [11]. These methods characterize the multi-dimensional structure of neural population activity based on how the activity of different neurons co-varies. Despite the growing use of dimensionality reduction in systems neuroscience, it is unclear whether results obtained using a limited number of neurons and trials are informative of the larger circuit from which the neurons are sampled [12]. Furthermore, since the connectivity structure of the neural population is typically unknown during *in vivo* recordings, it is unclear how these results relate to the underlying network structure. This paper addresses these gaps by applying dimensionality reduction to population activity from *in vivo* recordings and spiking network models.

While our ultimate goal is to understand the population activity structure of *in vivo* recordings, there are several important benefits of analyzing population activity generated by spiking network models. First, because we can sample as many neurons and trials as desired from a spiking network model, we can measure how the outputs of dimensionality reduction vary over a wide range of neuron and trial counts. This allows us to assess whether the results obtained using a limited number of neurons and trials are representative of the larger network. Second, we can assess how these scaling trends are influenced by the known underlying network structure. Third, we can study how the results are influenced by which neurons are sampled in the network. This paper utilizes these three benefits of spiking network models to develop a deeper intuition for the relationship between the outputs of dimensionality reduction and the underlying neural circuit.

Spiking network models that balance excitation and inhibition have been widely studied to understand the mechanisms underlying spike timing variability and correlated variability across neurons (e.g., [13–17]). Recent studies have introduced clustering structure and found that the single-neuron and pairwise statistics of these networks better capture the slow fluctuations in firing rate observed during *in vivo* recordings [17–19]. In this work, we focus on two types of spiking network models: clustered networks and classic balanced (non-clustered) networks.

To study single-trial population activity, we used factor analysis (FA), a linear dimensionality reduction method [2, 4, 20–22]. A key feature of FA is that it partitions spike count variability into a component that is shared among the recorded neurons and a component that is independent across neurons. From a statistical perspective, the shared component can be thought of as the co-fluctuations of the underlying firing rates of the recorded neurons, and the independent component can be thought of as the Poisson-like spiking variability of neurons [5, 23]. Whereas the independent component could be averaged away during downstream processing by pooling across neurons, the shared component can be particularly consequential for behavior [24, 25]. The partition between the shared and independent components can depend on which neurons are recorded. For example, a neuron might not co-vary with any of the recorded neurons, and instead co-vary with unrecorded neurons. By recording from more neurons, a larger proportion of a neuron’s spike count variability may be assigned to the shared component, and correspondingly less to the independent component.

In this work, we leveraged this separation of variability into shared and independent components to quantify two aspects of the population activity structure: (1) shared dimensionality, which is a measure of the complexity of the shared activity co-fluctuations, and (2) percent shared variance, which measures the prominence of the shared component in the spiking activity. These measures generalize the ideas behind spike count correlation [26], measured between pairs of neurons, to an entire population of neurons. In addition, we used FA to identify the characteristic ways in which the neurons co-fluctuate, known as the modes of population activity. The modes of population activity have provided insight about the neural basis of working memory [27], decision making [3, 28], motor preparation [29, 30], and learning [4].

We studied the scaling trends of shared dimensionality and percent shared variance with increasing numbers of neurons and trials. To perform this analysis we used spontaneous activity recorded in the primary visual cortex (V1) of anesthetized macaque monkeys and activity generated from non-clustered and clustered spiking network models. In addition, we assessed the effects of network structure on these metrics and found substantial differences in the scaling properties of the clustered and non-clustered networks, with the clustered network showing many similarities to the *in vivo* recordings. Furthermore, *in vivo* recordings from tens of neurons were sufficient to identify dominant modes of shared variability that generalized to recordings of larger numbers of neurons. Our results demonstrate how the outputs of dimensionality reduction depend on the amount of data and the underlying network structure, and provide support for the use of dimensionality reduction with current recording technologies (i.e., tens of neurons and hundreds of trials).

## Results

**Fig 1:**
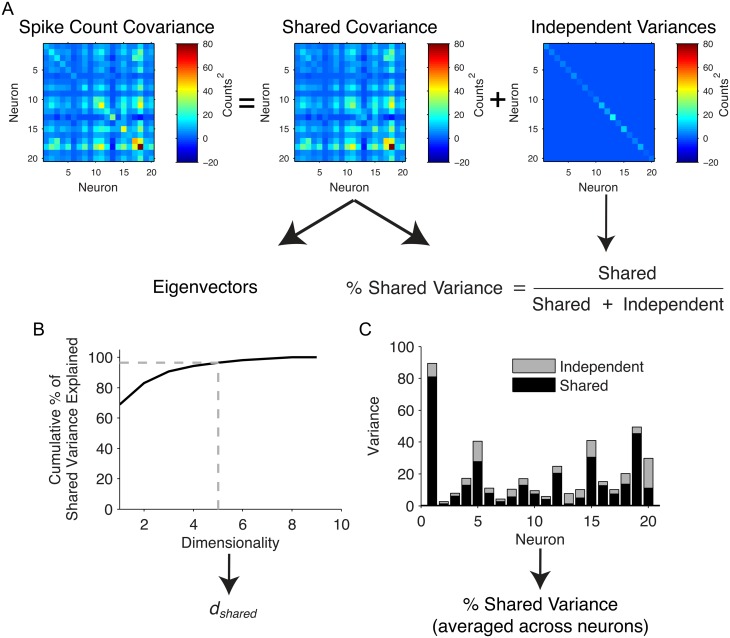
Calculation of shared dimensionality and percent shared variance. (A) Factor analysis partitions the spike count covariance matrix into shared and independent components. (B) Shared dimensionality (*d*_*shared*_) was defined as the number of eigenvectors (modes) required to explain 95% of shared variance. (C) The percent shared variance for an individual neuron is defined as the neuron’s shared variance divided by its total variance. We then averaged this across all neurons to obtain an overall percent shared variance.

A standard approach to studying pairwise relationships in populations of simultaneously recorded neurons over many trials is to compute the spike count covariance of the population (Fig 1A, left). To move beyond pairwise correlations to understand shared activity across the whole population, we first applied FA to partition the spike count covariance into a shared component and an independent component (Fig 1A, middle and right). We then computed two metrics to summarize population activity based on the shared component: shared dimensionality, or *d*_*shared*_ (Fig 1B), and percent shared variance (Fig 1C). The *d*_*shared*_ measures the complexity of the shared activity co-fluctuations, or the number of modes, among the neurons. For example, if *d*_*shared*_ equals one, then all of the shared variance in the population can be attributed to a single mode, whereas larger *d*_*shared*_ indicates the presence of multiple modes of shared variability. Percent shared variance measures the prominence of the shared component in the spiking activity, and is computed based on how much of each neuron’s activity co-varies with the activity of at least one other recorded neuron.

Before studying how *d*_*shared*_ and percent shared variance scale with the number of neurons and trials, it is important to recognize that these two measures are distinct. To see this, consider a population of neurons modulated by a common multiplicative gain factor that accounts for a large portion of the variance [31, 32]. In this case, *d*_*shared*_ would be one, and the percent shared variance would be high. On the other hand, suppose that a population is grouped into pairs of neurons, where each pair is modulated by a distinct multiplicative gain factor that accounts for a small portion of total variance. In this scenario, *d*_*shared*_ would be high (roughly equal to half the number of neurons in the population), and the percent shared variance would be low. Similar scenarios can be imagined that result in low *d*_*shared*_ and low percent shared variance or high *d*_*shared*_ and high percent shared variance. These scenarios show that *d*_*shared*_ and percent shared variance do not necessarily change together.

**Fig 2:**
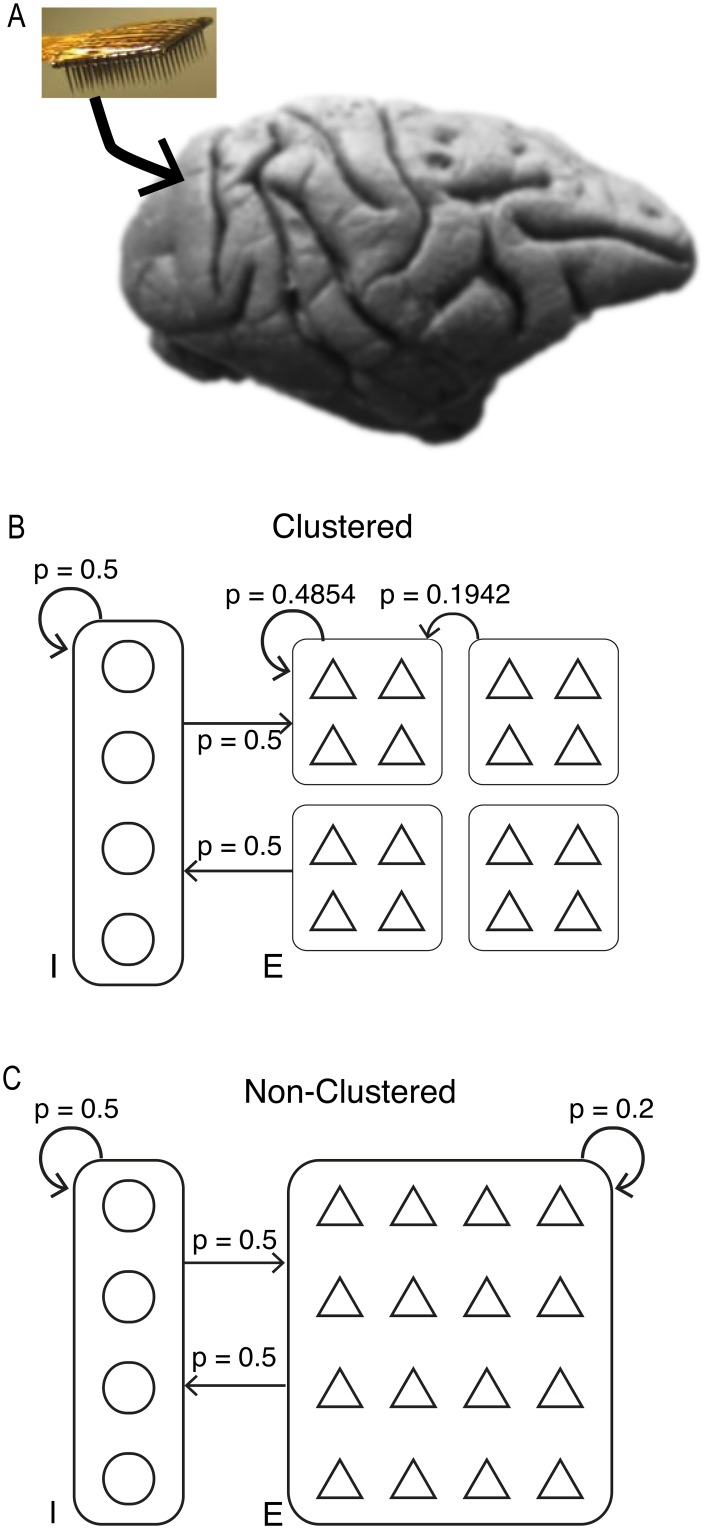
Neural populations and network models. (A) Neural activity was recorded using a Utah array implanted in V1 of macaque monkeys during presentation of an isoluminant gray screen. (B) Clustered network consisted of 4000 excitatory neurons grouped into 50 clusters of 80 neurons. Triangles represent excitatory neurons and circles represent inhibitory neurons. Clusters had high within-cluster connection probability relative to between-cluster connection probability. Connection probabilities between excitatory and inhibitory neurons indicated above corresponding arrow. (C) Non-clustered network consisted of 4000 excitatory neurons with homogeneous connection probabilities.

Below, we first assess the *d*_*shared*_ and percent shared variance of *in vivo* recordings while varying neuron and trial counts (Fig 2A). Then we apply the same analyses to spike counts generated from clustered (Fig 2B) and non-clustered (Fig 2C) spiking network models, allowing us to go beyond the range of neurons and trials available in the *in vivo* recordings. We perform these analyses on spontaneous neural activity. In the case of the *in vivo* recordings, spontaneous activity refers to activity recorded during the presentation of an isoluminant grey screen. In the spiking network models, spontaneous activity refers to the lack of dynamic external inputs to the network.

### Varying neuron and trial count for *in vivo* neural recordings

**Fig 3:**
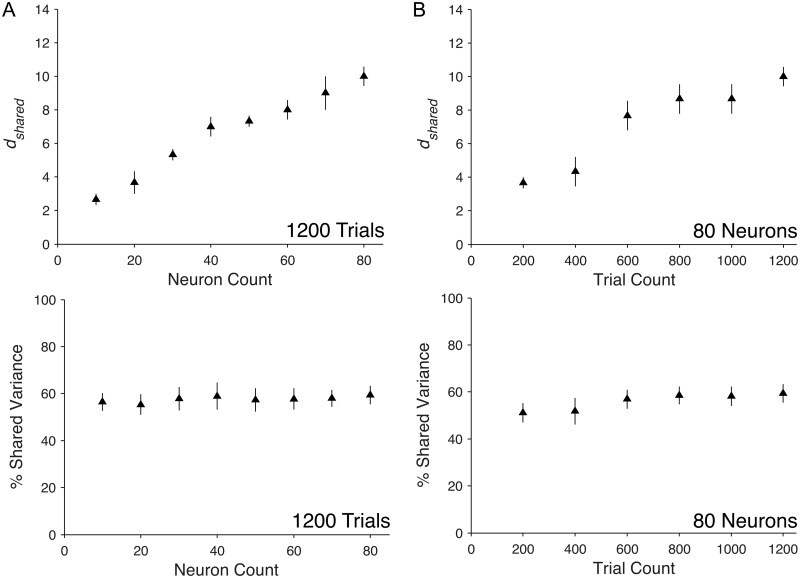
Scaling properties of shared dimensionality and percent shared variance with neuron and trial count in V1 recordings. The *d*_*shared*_ and percent shared variance over a range of (A) neuron counts and (B) trial counts from population activity recorded in V1. Each triangle represents the mean across single samples from each of three arrays. Error bars represent one standard error across the three arrays.

We first studied how *d*_*shared*_ and percent shared variance scale with neuron count for *in vivo* recordings. To do this, we applied FA to spontaneous activity recorded in primary visual cortex (V1) of anesthetized macaques. We binned neural activity into 1-second epochs, where each bin is referred to as a ‘trial’. Thus, the number of trials is equivalent to the recording time (in seconds). We sampled increasing numbers of neurons or trials from the recorded population activity, and measured *d*_*shared*_ and percent shared variance for each neuron or trial count. We expected *d*_*shared*_ and percent shared variance to either saturate or to increase with increasing neuron or trial count. Saturating *d*_*shared*_ would suggest that we have identified all of the modes for the network (or networks) sampled by the recording electrodes and increasing *d*_*shared*_ would suggest that additional modes are being revealed by monitoring additional neurons or trials. We found that *d*_*shared*_ increased with neuron count (Fig 3A, Top), while percent shared variance remained stable with increasing neuron count (Fig 3A, Bottom). Similarly, additional trials resulted in increasing *d*_*shared*_ and stable percent shared variance (Fig 3B). These scaling trends in *d*_*shared*_ and percent shared variance remained the same for spike count bins ranging from 200 ms to 1 second (S1 Fig). We also found that not taking into account the sequential nature of the time bins when using factor analysis was reasonable for 1-second bins (S2 Fig). Together these results demonstrate that, within the range of neurons and trials available from our recordings, additional neurons and trials allow us to identify additional shared dimensions. This implies that we have not sampled enough neurons or trials to identify all of the modes of shared variability. However, given the stable percent shared variance observed in Fig 3A and 3B (bottom panels), the results suggest that the shared component is dominated by the first few modes and that additional modes do not explain substantial shared variance. This is supported by analyses in the next section.

### Modes of shared variability for *in vivo* neural recordings

Recent studies have explored how different modes of population activity are used during different task epochs [28, 29], during learning [4], and after perturbations [30], as well as to encode different types of information [3, 27]. It is currently unclear how the modes identified with a limited sampling of neurons relate to those identified from increasingly larger samplings. We studied this question by measuring (1) shifts in the subspaces spanned by the dominant modes and (2) changes in percent shared variance along each mode as neurons are added to the analysis.

**Fig 4:**
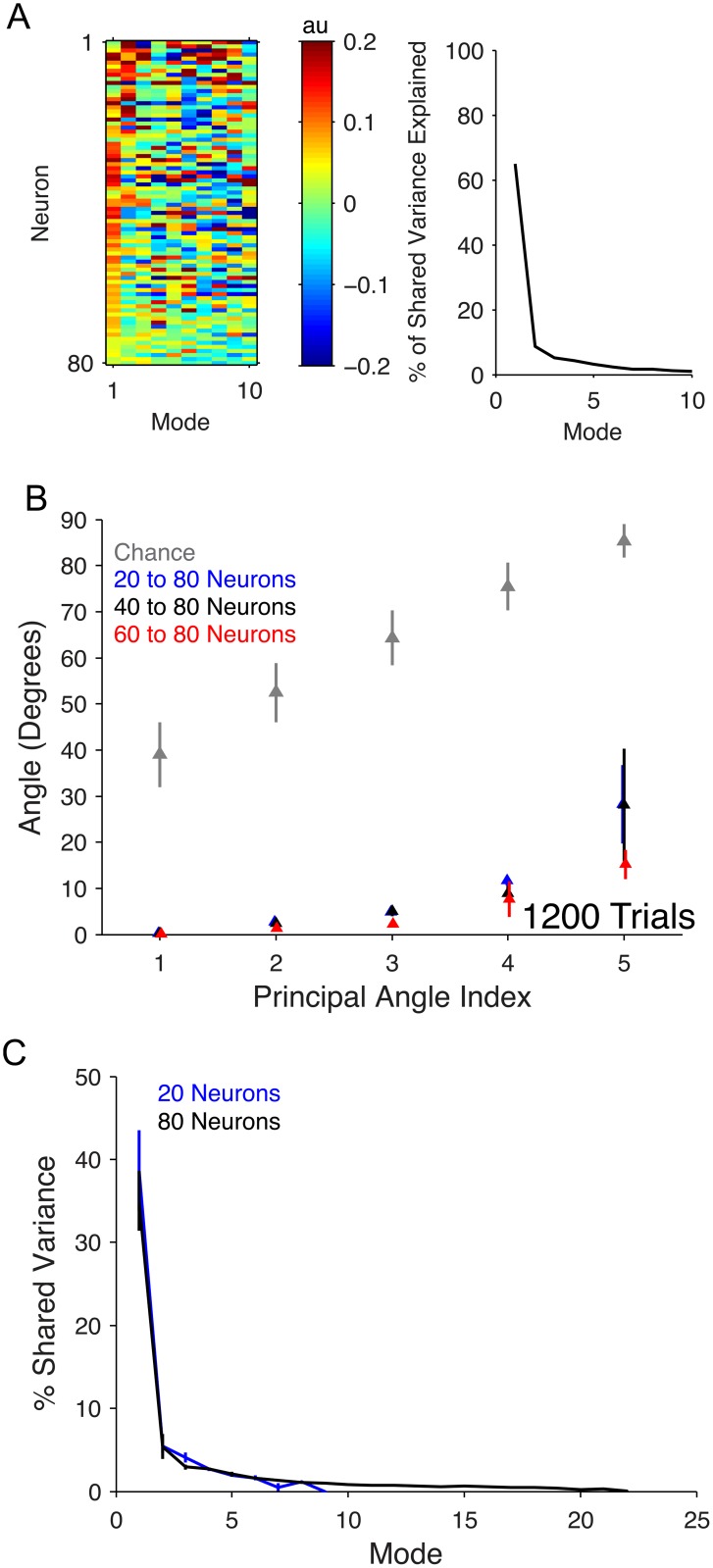
Modes of shared variability in V1 recordings. (A) Left: Modes of *in vivo* recordings. Each column of the heatmap is an eigenvector of the shared covariance matrix computed from a set of 80 neurons and 1200 trials. Columns are ordered with modes explaining the most shared variance on the left. Neurons (rows) are ordered with highest mean firing rate at the top to lowest mean firing rate at the bottom. Right: Percent of shared variance explained by each mode. Plot shows mean across three arrays. Trends were similar in each of the three arrays. (B) Principal angles between modes in *in vivo* recordings for 20- (black), 40- (blue), or 60-neuron (red) analyses and corresponding neurons from 80-neuron analyses. Modes were identified by computing the eigenvectors of the shared covariances corresponding to neurons from the 20-neuron set. Triangles and error bars represent mean and standard error across the three arrays, respectively. Grey triangles represent principal angles (mean ± one standard deviation) between random 20-dimensional vectors. (C) Percent shared variance along each mode in the clustered network for 20-neuron analyses (blue) and 80-neuron analyses (black) used in (B). Note that the maximum number of modes (across the three arrays) in the 20-neuron sets was 9 and the maximum number of modes in the 80-neuron sets was 22. The recordings from each array had at least 5 modes. Curves and error bars represent mean percent shared variance and standard error for each mode across single samples from each of three arrays.

We first examined the modes for the *in vivo* recordings (Fig 4A left panel), ordered from most dominant (i.e., explaining the largest amount of shared variance) to least dominant. Consistent with previous work [31–34], the most dominant mode (left-most column in Fig 4A) comprised many entries of the same sign, implying that a large portion of shared activity resulted from many neurons increasing and decreasing their activity together. This mode accounted for over 60% of the shared variability (Fig 4A right panel), and there were other modes representing more complex interactions that also accounted for a substantial proportion of shared variability.

To measure how modes of shared variability from *in vivo* recordings changed direction in the population activity space with increasing neuron count, we performed the following procedure. We first sampled sets of 80 excitatory neurons from each array and then subsampled 20, 40, or 60 neurons from each 80-neuron set. We sampled such that the 40-neuron sample was a subset of the 60-neuron sample and that the 20-neuron sample was a subset of the 40-neuron sample, ensuring that all sets contained the 20-neuron sample. We then identified the five most dominant modes in each of the 20-, 40-, 60-, or 80-neuron sets. We measured the principal angles between modes from the subsampled sets and modes from the 80-neuron set based on the entries in each mode corresponding to the neurons in the 20-neuron set. Smaller principal angles indicated greater similarity between modes. Fig 4B shows that the most dominant modes remained largely unchanged as neuron count increased. By definition, the angles increased with principal angle index because angles were computed beginning with the smallest possible angle between sets of modes. Percent shared variance along each mode also remained stable with increasing neuron count (Fig 4C). Note that the quantity plotted in Fig 4A (right panel) is related to, but different from, the quantity plotted in Fig 4C. Whereas Fig 4A (right panel) considers only the shared variability, Fig 4C assesses how much of the overall spike count variability is assigned to the shared component (as in Fig 3, see Methods for details). Overall, our study of the modes from *in vivo* recordings revealed that a few dominant modes explained most of the shared variance and that these dominant modes remained stable as we added neurons to the analysis.

### Varying neuron and trial count for network models within the experimental regime

In the previous sections, we identified trends in *d*_*shared*_ and percent shared variance using *in vivo* recordings. Several experimental constraints limit the types of questions we can ask using *in vivo* recordings. First, we are limited in the number of neurons and the number of trials that are recorded. Second, in most experiments, we have no knowledge of the connectivity structure of the underlying network and cannot relate properties of the population activity to network structure. In this section we overcome these constraints by analyzing activity obtained from network models.

We consider recurrent spiking network models with distinct excitatory and inhibitory populations whose synaptic interactions are dynamically balanced [13, 14]. In particular, we focus on two subclasses of this model: one where excitatory neurons are grouped into clusters that have a high connection probability (clustered network) and one where the excitatory population has homogeneous connectivity (non-clustered network). Both the clustered and non-clustered networks have been shown to capture variability in spike timing [14, 17]. Clustered networks have also been shown to demonstrate slow fluctuations in firing rate [17] consistent with *in vivo* recordings [20, 35, 36].

In the particular clustered network studied here, each cluster resembles a bistable unit with low and high activity states that lead neurons in the same cluster to change their activity together. We expected to identify dimensions that reflected these co-fluctuations within clusters, resulting in *d*_*shared*_ bounded by the number of clusters (i.e., 50 dimensions) and high percent shared variance. In contrast, the non-clustered network lacks the highly correlated activity seen in the clustered network [13, 14, 17], and so we expected to see little or no shared variance. Note that no shared variance would result in both percent shared variance and *d*_*shared*_ being zero. Small amounts of shared variance relative to total variance would result in low percent shared variance and either low or high *d*_*shared*_ depending on the multi-dimensional structure of the shared variance.

**Fig 5:**
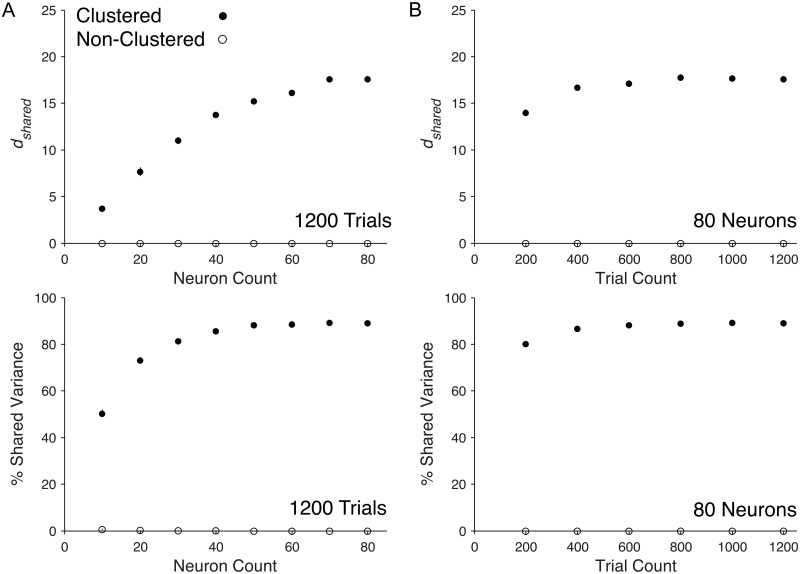
Scaling properties of shared dimensionality and percent shared variance with neuron and trial count in spiking network models. The *d*_*shared*_ and percent shared variance over a range of (A) neuron counts and (B) trial counts from clustered (filled circles) and non-clustered (open circles) networks. Circles represent mean across the five non-overlapping sets of neurons and five non-overlapping sets of trials (25 total sets) and error bars represent standard error across all sets. Standard error was generally very small and therefore error bars are not visible for most data points.

To test how clustered connectivity affects the population activity structure and to understand how the population-level metrics scale with the number of neurons and trials, we performed the following analysis. We applied FA to spike counts, from non-clustered and clustered network simulations. Each spike count was taken in a 1-second bin of simulation time, which we refer to as a ‘trial’ in analogy to physiological recordings. We then increased the neuron count, as we did in Fig 3 for the *in vivo* recordings, with the number of trials fixed at 1200 to match the analyses shown in Fig 3A. We observed increasing *d*_*shared*_ with neuron count in the clustered network and a *d*_*shared*_ of zero for all neuron counts in the non-clustered network (Fig 5A, Top). The percent shared variance for the clustered network increased with neuron count and saturated at approximately 90% (Fig 5A, Bottom). In contrast, the non-clustered network showed zero percent shared variance at all neuron counts. In other words, in the range of trials and neurons studied, FA could not identify any shared population-level structure in the non-clustered network. These results agree with our predictions, namely non-zero *d*_*shared*_ and high percent shared variance in the clustered network and zero *d*_*shared*_ and percent shared variance in the non-clustered network.

We next investigated how *d*_*shared*_ and percent shared variance change for an increasing number of trials, with the number of model neurons fixed at 80 to match the analyses shown in Fig 3B. We anticipated that *d*_*shared*_ and percent shared variance would increase to a saturation point after which enough trials would be available to reliably identify all of the modes of shared variability. In the clustered network, we observed that *d*_*shared*_ (Fig 5B, Top) and percent shared variance (Fig 5B, Bottom) initially increased and then saturated, indicating that fewer than 1200 trials were needed to characterize *d*_*shared*_ and percent shared variance for 80 neurons sampled from the clustered network. In the non-clustered network, we observed zero *d*_*shared*_ and percent shared variance for all trial counts. Therefore, of the two networks studied, only the clustered network demonstrated population-level shared structure within the range of trials obtained in the *in vivo* recording.

Comparing the model network results (Fig 5) with the experimental results (Fig 3) obtained from equal numbers of neuron and trials, we observed similar trends in the clustered network and *in vivo* recordings. In both cases we observed increasing *d*_*shared*_ and saturating percent shared variance with neuron and trial count. Note that we did not tune network parameters (e.g., firing rates, number of clusters, etc.) in the clustered network to match the *in vivo* recordings and, therefore, we did not expect the magnitudes of *d*_*shared*_ or percent shared variance to match in the two cases. However, the trends of increasing *d*_*shared*_ with neuron and trial count accompanied by stable percent shared variance suggest that, in both cases, the population activity is largely governed by a few dominant modes that are well characterized within the range of neurons and trials obtainable with current recording technology.

### Varying neuron and trial count for network models outside of experimental regime

**Fig 6:**
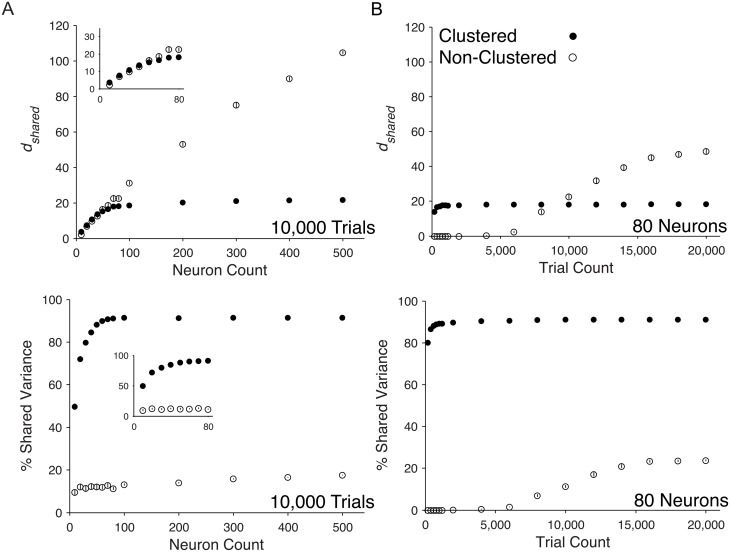
Scaling properties of shared dimensionality and percent shared variance with large neuron and trial counts in spiking network models. The *d*_*shared*_ and percent shared variance over a range of (A) neuron counts and (B) trial counts from clustered (filled circles) and non-clustered (open circles) networks. Insets zoom in on range of neurons used in *in vivo* recordings in Fig 3. Circles represent mean across the five non-overlapping sets of neurons and five non-overlapping sets of trials (25 total sets) and error bars represent standard error across all sets. Standard error was generally very small and therefore error bars are not visible for most data points.

To better understand how the outputs of dimensionality reduction for limited sampling reflect larger portions of the network, we investigated how the trends from Fig 5 continued for larger numbers of neurons and trials. We first varied the number of neurons in the analysis up to 500 neurons. This required us to increase the number of trials from 1200 to 10,000 trials in order to fit the larger number of parameters in the FA model. We found that *d*_*shared*_ in the clustered network saturated with roughly 100 neurons, whereas *d*_*shared*_ in the non-clustered network continued to increase with neuron count (Fig 6A, Top). In both networks, the percent shared variance remained stable with additional neurons, but the clustered network had higher shared variance than the non-clustered network (Fig 6A, Bottom). Within the experiment regime of neuron counts (10 to 80 neurons) we found non-zero *d*_*shared*_ and percent shared variance for both networks (Fig 6A, Inset). Overall, in the clustered network, we observed saturation in *d*_*shared*_ and percent shared variance with few neurons relative to the network size. This likely stemmed from the fact that neurons from the same cluster varied together. Therefore, we were able to identify the majority of shared variance once multiple neurons were sampled from most clusters. That result contrasts with our observation of increasing dimensionality and low shared variance in the non-clustered network, which lacks modes describing activity from groups of co-varying neurons. It is therefore likely that we identified many modes that each explain small amounts of variability. We investigate this in greater detail below.

To study the effects of large trial count on population-level metrics, we computed *d*_*shared*_ and percent shared variance for 80 neurons while varying the trial count up to 20,000 (Fig 6B). The non-clustered network had no identifiable shared population activity structure when the trial count was less than 5,000, consistent with Fig 3; however, with 5,000 or more trials, we identified non-zero *d*_*shared*_. It is clear from this result that trial counts within the experimental regime were insufficient to identify shared dimensions, but that additional trials revealed shared dimensions. Percent shared variance followed a similar trend, with zero percent shared variance below 5,000 trials, as expected given zero *d*_*shared*_. These results show that many more trials were required to identify the small amounts of shared variability in the non-clustered network compared to the clustered network.

The above analyses showed substantial differences between the two model networks. In the clustered network, the shared population activity structure was salient (approximately 90% of the raw spike count variability was shared among neurons) and defined by a small number of modes (approximately 20 modes), all of which could be identified using a modest number of neurons and trials. In contrast, for the non-clustered network, the shared population activity was more subtle (approximately 20% of the raw spike count variability was shared among neurons), distributed across many modes, and required large numbers of trials to identify.

### Varying the number of clusters represented in sampled neurons

**Fig 7:**
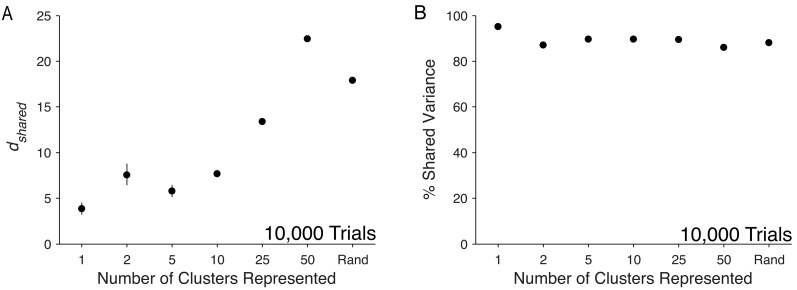
Influence of cluster representation on shared dimensionality and percent shared variance in the clustered network. Dependence of (A) *d*_*shared*_ and (B) percent shared variance on cluster representation in the set of sampled neurons. Analyses were performed for 50 neurons with 10,000 trials. ‘Rand’ indicates random sampling over all excitatory neurons. Circles represent mean across five non-overlapping sets of neurons and five non-overlapping sets of trials (25 total sets) of a single network with clustered structure. Error bars represent standard error across all sets.

So far we have sampled neurons at random from the model networks. However, in our *in vivo* recordings, we sampled from a spatially restricted population of neurons. When analyzing a sampling of neurons from a network, it is unclear how the particular neurons that are sampled influence *d*_*shared*_ and percent shared variance. To investigate the effects of non-random sampling procedures, we varied the number of clusters represented in a 50-neuron set. We found that *d*_*shared*_ generally increased with cluster representation (Fig 7A). Interestingly, *d*_*shared*_ exceeded cluster representation for low cluster counts, likely representing less dominant modes that are washed out when more clusters are represented. For the 50-cluster case, *d*_*shared*_ was 22.5 ± 0.17 (mean ± standard error), roughly equal to the saturation value for the clustered network of 21.8 ± 0.25 (mean ± standard error, 500-neuron, filled data point in Fig 6A). Unlike *d*_*shared*_, percent shared variance remained stable across a wide range of cluster representation (Fig 7B), even in the most extreme cases of sampling all neurons from a single cluster (one cluster represented) and sampling one neuron from each cluster (50 clusters represented).

In our analyses, the distribution of samples across the network influenced the observed *d*_*shared*_, with broader distributions (i.e., with more clusters being represented in the sample) better characterizing the overall network. With only 50 carefully chosen neurons, we obtained the saturation *d*_*shared*_ and percent shared variance shown in Fig 6. For *in vivo* networks, our lack of information about the underlying connectivity of the network prevents us from knowing exactly how we should sample to minimize the number of neurons required to fully characterize the population activity structure of the network. However, our results suggest that tailored sampling procedures may allow characterization of shared variability with fewer neurons than random sampling.

### Modes of shared variability for network models

**Fig 8:**
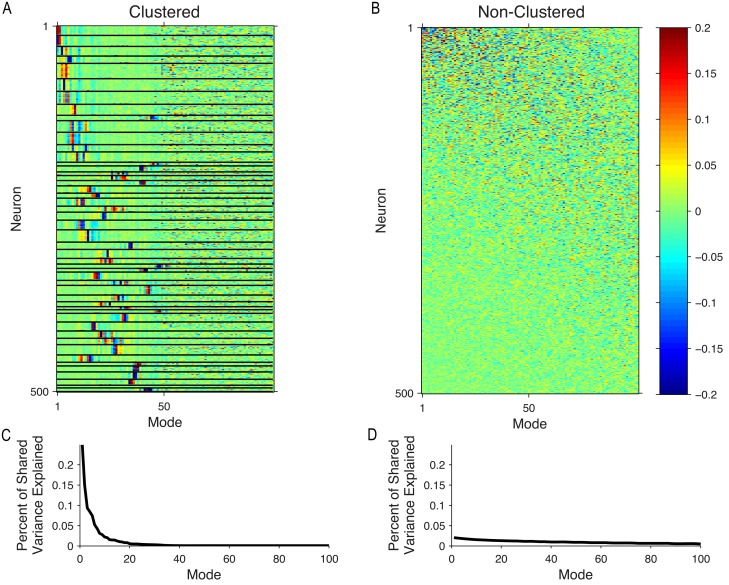
Modes of shared variability for spiking network models. (A) Left: Modes of clustered network. Each column of the heatmap is an eigenvector of the shared covariance matrix computed from a set of 500 neurons and 10,000 trials. Columns are ordered with modes explaining the most shared variance on the left. Neurons (rows) are ordered by cluster (black lines indicate cluster boundaries), sorted with the highest mean firing rate clusters at the top. Note that due to random sampling there are an unequal number of neurons in each cluster. (B) Modes of non-clustered network. Same conventions as in (A), except rows are ordered by firing rate of individual neurons, with the highest mean firing rate at the top. The number of dimensions that maximized the cross-validated data likelihood was 100 in (A) and 110 in (B). (C) Percent of shared variance explained by each mode in (A). (D) Percent of shared variance explained by each mode in (B).

As we did with the *in vivo* recordings (Fig 4), we also examined the modes of shared variability for the model networks. In the top 50 modes of the clustered network (Fig 8A, rows sorted by cluster identity and mean cluster firing rate), the entries corresponding to same-cluster neurons tended to have similar values. Thus these modes described same-cluster neurons increasing or decreasing their activity together. Since the modes were ordered by dominance (i.e., columns ordered by amount of shared variance explained), this indicates that the dominant interactions in the clustered network are those between clusters. The modes beyond the 50^th^ mode did not reflect the cluster identities of the neurons, but instead described more subtle interactions between neurons both within and across clusters. Additionally, neurons or clusters of neurons with higher mean firing rates tended to be involved in more dominant modes in both model networks (Fig 8A and 8B). This is reasonable because neurons with higher firing rates tend to have higher variance [37], and are therefore capable of covarying more strongly with other neurons (i.e., show higher activity covariance) than neurons with lower firing rates. In contrast to the clustered network, there was no apparent clustering in the mode entries for the non-clustered network (Fig 8B), as one would expect from the random uniform connectivity of the network.

Comparing the modes for the model networks (Fig 8A and 8B) to those for the *in vivo* recordings (Fig 4A), neither model network reproduced the first dominant mode of the *in vivo* recordings, which described all neurons increasing and decreasing their activity together. We further asked whether it would be possible to reorder the neurons from the *in vivo* recordings (Fig 4A) to obtain clustering structure as shown in Fig 8A for the clustered network. Using the k-means algorithm to try to identify similar rows of the modes matrix, we did not find clear clustering structure in the *in vivo* recordings (S3 Fig, also see Discussion).

Each of the modes in Fig 8A and 8B describe some percentage of the overall shared variance. A small number of dominant modes explained a large proportion of the shared variance in the clustered network (Fig 8C), whereas most of the modes in the non-clustered network explained similar amounts of shared variance (Fig 8D). We summarized these curves (Fig 8C and 8D) using *d*_*shared*_, defined as the number of modes needed to explain 95% of the shared variance (see Methods). For a representative sample of neurons and trials from the clustered network, only 20 modes were needed to describe 95% of the shared variance (Fig 8C, consistent with Fig 6A for 500 neurons), whereas 99 modes were needed in the non-clustered network (Fig 8D, consistent with Fig 6A for 500 neurons). Although one might initially expect *d*_*shared*_ to equal the number of clusters (50) in the clustered network, we found that *d*_*shared*_ was 20 because the top 20 modes were sufficient for explaining 95% of the shared variance.

**Fig 9:**
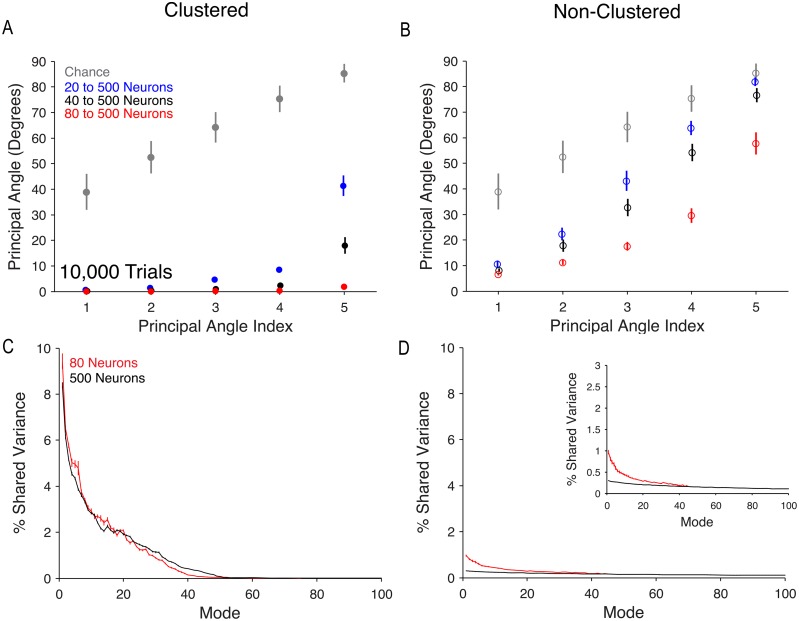
Stability of modes of shared variability in network models. (A) Principal angles between top five modes in clustered network for 20- (blue), 40- (black), or 80-neuron (red) analyses and corresponding neurons from 500-neuron analyses. Modes were identified by computing the eigenvectors of the shared covariances corresponding to neurons from the 20-neuron set. Plots show mean and standard error across 25 sets of 500 neurons and 10,000 trials. Grey circles represent principal angles (mean ± one standard deviation) between random 20-dimensional vectors. (B) Principal angles between modes in the non-clustered network. Same conventions as in (A). (C) Percent shared variance along each mode in the clustered network for 80-neuron analyses (red) and 500-neuron analyses (black) shown in (A). The maximum number of modes across the 25 sets was 75 for the 80-neuron analysis and 130 for the 500-neuron analysis. The two curves were nearly identical between modes 50 and 75 and therefore only the first 100 modes are shown. Curves represent mean percent shared variance for each mode across 25 sets. Error bars show standard error computed across the 25 sets. (D) Percent shared variance along each mode in the non-clustered network for the 80-neuron analyses (red) and the 500-neuron analyses (black) used in (B). Same conventions as in (C). The maximum number of modes across the 25 sets was 45 in the 80-neuron analysis and 130 for the 500-neuron analysis. Inset shows zoomed in vertical axis.

We then assessed how the modes of shared variability changed direction in the multi-dimensional population activity space with increasing neuron count, using the same procedure as with the *in vivo* recordings (Fig 4B). We found that, as neuron count increased, principal angles between the modes from the subsampled population and the modes from the 500-neuron population decreased in both networks (Fig 9A and 9B), indicating that the modes became more similar to those of the 500-neuron set as neuron count increased. This implies that sampling additional neurons provides a better characterization of the modes. In the clustered network, the principal angles decreased to near zero in the 80-neuron set (Fig 9A), demonstrating that the first five modes were nearly identical in the 80-neuron and 500-neuron sets. However, in the non-clustered network, principal angles remained relatively large for all sets (Fig 9B). These results show that, with as few as 80 neurons, we obtain an accurate estimate of the modes of shared variability in the wider network in the clustered case, but not the non-clustered case.

Analyzing the modes of shared variability allows us to better understand trends observed in Fig 6A. Typically, one would expect percent shared variance to increase when *d*_*shared*_ increases because each dimension explains some amount of (positive) shared variance. However, for the non-clustered network, we found that *d*_*shared*_ increased without an associated increase in percent shared variance. This can be understood by the fact that the dominant modes changed as more neurons were added to the analysis (Fig 9B). As a result, the amount of shared variance explained by the leading modes could decrease as more modes are identified. We assessed this by partitioning the overall percent shared variance in Fig 6A into a percent shared variance along each mode and examining how the distribution of percent shared variance across the modes changed with additional neurons. In the clustered network, we found that percent shared variance was very similar between the 80- and 500-neuron sets (Fig 9C), with percent shared variance in the top five modes (the same modes used in Fig 9A) dropping only 9.22 ± 1.70% (mean ± standard error). In contrast, for the non-clustered network, percent shared variance dropped 60.7 ± 2.07%(mean ± standard error) (Fig 9D) in the top five modes (the same modes used in Fig 9B). Thus, there is a shift in percent shared variance from dominant to less dominant modes in the non-clustered network as neurons are added, which explains how it is possible for *d*_*shared*_ to increase without an associated increase in percent shared variance.

For the *in vivo* recordings, we also see that *d*_*shared*_ increases without an associated increase in percent shared variance (Fig 3A). However, this occurs for a different reason than for the non-clustered network. As neurons are added to the *in vivo* analysis, the dominant modes tend to be stable (Fig 4B), so we do not see the same shift in percent shared variance from dominant to less dominant modes (Fig 4C, 17.7% ± 2.3 drop in percent variance in the top five modes) as in the non-clustered network. Furthermore, the additional modes identified with more neurons explain only small amounts of shared variance relative to the dominant modes (Fig 4C). Thus, the percent shared variance appears not to increase for the *in vivo* recordings because the additional shared variance contributed by newly identified dimensions is small.

In summary, for the clustered network, the dominant modes of shared variability among the original neurons remained stable as neurons were added to the analysis. In contrast, the non-clustered network modes changed as neurons were added to the analysis and tended to become less dominant (i.e., the percent shared variance along those modes decreased). The results shown here for the clustered network are largely consistent with the results for *in vivo* recordings (Fig 4B and 4C). The similarities between the clustered network and *in vivo* recordings remained true when we matched number of neurons and trials for the clustered network to *in vivo* recordings (S4 Fig).

## Discussion

In this study, we used V1 recordings and spiking network models to understand how the results obtained using dimensionality reduction methods generalize to recordings with larger numbers of neurons and trials, as well as how these results relate to the underlying network structure. We found that recordings of tens of neurons and hundreds of trials were sufficient to identify the dominant modes of shared variability in both *in vivo* recordings and a spiking network model with clustered connectivity. Comparing spiking network models, we found that scaling properties differed in non-clustered and clustered networks and that *in vivo* recordings were more consistent with the clustered network. These findings can help guide the interpretation of dimensionality reduction analyses in terms of limited neuron and trial sampling and underlying network structure.

We focused on variability that is shared among simultaneously-recorded neurons. Shared variability has been widely studied due to its implications for the amount of information that is encoded by a population of neurons [25]. For the same population of neurons, the dimensionality computed using the raw (spike count) covariability can be substantially different from that computed using the shared covariability. To see this, consider a population of independent neurons. As the number of neurons in the analysis grows, the dimensionality based on the raw covariability would increase, whereas the dimensionality based on the shared covariability (i.e., *d*_*shared*_) would remain at zero because independent neurons have no shared variance.

We used FA to partition the raw covariance matrix into shared and independent components and measured the dimensionality of the shared component [5, 23]. By contrast, principal components analysis (PCA), a standard dimensionality reduction method, applied to spike counts measures dimensionality of the raw covariability. Recently, Mazzucato et al. used PCA to examine the dimensionality of 3 to 9 neurons recorded simultaneously in rat gustatory cortex [19]. Despite the difference in methods used to compute dimensionality, they also found that dimensionality increases with neuron and trial count in *in vivo* recordings and spiking network models. Our use of FA to isolate the shared and independent components provides two important insights. First, we are able to assess the scaling trends of the dimensionality of the shared component in isolation. Relative to independent variability, shared variability is more difficult to average away within the network and is therefore more likely to influence downstream processing. Our dimensionality measurement indicates the richness of this shared aspect of the population activity. Second, we can measure the percent of the overall variance that is shared across neurons, which provides context to the dimensionality metric. For example, in the non-clustered network (Fig 6A, Top), given many trials and neurons, we identified many shared dimensions. However, these dimensions represented only a small fraction of the overall variance (Fig 6A, Bottom). By contrast, the clustered network exhibited fewer dimensions, but those dimensions represented a large fraction of the overall variance (Fig 6A). These results suggest that FA provides a more nuanced characterization of single-trial population activity than PCA.

In this work, we studied spontaneous activity during *in vivo* recordings and in spiking network models. Our study could be extended to scaling trends in evoked activity, in which visual stimuli are presented during the V1 recordings and non-zero inputs are used in the spiking network models. Previous studies have found that shared variance tends to decrease after stimulus presentation [20, 35, 38–40] and that the scaling properties of PCA dimensionality change after stimulus presentation [19]. However, under certain conditions, the population activity patterns expressed in spontaneous activity can resemble those expressed in evoked activity [9, 41]. Studying the scaling properties of shared dimensionality and percent shared variance using evoked activity would allow us to better interpret such results in the context of limited neuron and trial sampling.

We focused on trends in shared dimensionality and percent shared variance. Specific shared dimensionality and percent shared variance values obtained for the model networks likely depend on model parameters, including the number of clusters, the synaptic weights, and the probability of synaptic connection. We used the parameters described in [16], and we did not attempt to adjust parameters to make results match those found in experimental data. An interesting avenue for future work would be to understand the trade-offs among the different model parameters necessary to reproduce the absolute levels of shared dimensionality and percent shared variance measured for the *in vivo* recordings.

While many existing models reflect various aspects of neural activity, we studied two balanced spiking network models, which can be viewed as representing the two ends of a connectivity spectrum. At one end is the classic balanced network with homogeneous connectivity which has been studied for decades (i.e., the non-clustered network) [14, 15]. At the other end is a balanced network in which each excitatory neuron belongs to a particular cluster and there is high within-cluster connectivity (i.e., the clustered network) [17–19]. Although neither of these model networks is a perfect match with cortical networks, both model networks have been shown to mimic some single-neuron and pairwise spiking statistics measured in cortical neurons [14, 15, 17, 19]. Model networks which bridge these two ends of the spectrum are currently under development. Examples include networks with spatially dependent connectivity [42] and explicit stimulus tuning structure [43]. Analysis methods similar to those used here can be applied to study the population activity structure in those networks.

Comparisons between network models and *in vivo* recordings are usually made using aggregate single-neuron and pairwise statistics, such as mean firing rate, Fano factor, or Pearson correlation [13, 14, 17]. To move beyond single-neuron and pairwise statistics, the present work illustrates how multi-dimensional population statistics can be used to compare model networks and *in vivo* recordings. This approach has been adopted by several recent studies [3, 18, 19, 44] and can reveal discrepancies in the multi-dimensional activity patterns produced by model networks compared to biological networks of neurons. For example, the dominant mode of the *in vivo* recordings represented many neurons increasing and decreasing their activity together (Fig 4B, most elements in left-most column of the mode matrix are of the same sign). However, neither the clustered nor the non-clustered model reproduced this activity pattern in their dominant mode (Fig 8A and 8B). Such observations can guide the development of future network models.

Recent developments in neural recording technology are making it feasible to record from orders of magnitude more neurons simultaneously than what is currently possible (e.g., [45]). Thus it may soon be possible to analyze population activity for larger neuron counts from *in vivo* recordings. Furthermore, recent work has demonstrated the ability to access underlying network connectivity during *in vivo* recordings, an advance that may make it possible to determine the effects of connectivity structure on population activity [46, 47]. However, the number of trials available for studying population activity is still limited by various experimental constraints, such as an animal’s satiation or recording stability. To increase trial counts, it may be possible to combine data across multiple sessions by identifying the same neurons across multiple sessions [48–50] or by applying novel statistical methods [51–53].

## Materials and Methods

### Ethics statement

All experimental procedures followed guidelines approved by the Institutional Animal Care and Use Committees of the Albert Einstein College of Medicine at Yeshiva University and New York University, and were in full compliance with the guidelines set forth in the US Public Health Service Guide for the Care and Use of Laboratory Animals.

### Neural recordings

Details of the neural recordings were reported previously [38, 54]. Briefly, we recorded from primary visual cortex of anesthetized, paralyzed male macaque monkeys. We maintained anesthesia throughout the experiments, typically 5-7 days, with a continuous intravenous infusion of sufentanil citrate (6-18 *μ*g/kg/hr). Eye movements were minimized with a continuous intravenous infusion of vecuronium bromide (100-150 *μ*g/kg/hr).

We implanted multi-electrode arrays in primary visual cortex (V1) in three hemispheres of two anesthetized macaque monkeys. We recorded spontaneous activity for 20–30 minutes while a uniform gray screen was displayed on a computer monitor in front of the animal. Recorded waveform segments were sorted off-line using a competitive mixture decomposition method [55], after which waveform sorting for each electrode was refined by hand with custom time-amplitude window discrimination software taking into account waveform shape and inter-spike interval distribution. Signal-to-noise ratio (SNR) was then computed as the ratio of the average waveform amplitude to the standard deviation across waveforms [56]. Units with SNR below 1.5 and average spike counts less than one spike per second were excluded from the analyses, yielding a mixture of single- and multi-units, with median SNR of 2.74, 2.39, and 2.30 in the three arrays. The total number of units for each array was 118, 88, and 82 units. We randomly selected 80 units from each array to facilitate comparison between arrays. We then divided the neural activity into 1-second epochs. We refer to each of those 1-second epochs as a “trial” throughout this work.

### Spiking network simulations

Network simulations were performed using the same parameters as described in [17]. Briefly, we constructed a network of simulated neurons consisting of 4000 excitatory and 1000 inhibitory neurons. The voltage, *V*, for each neuron was modeled with the differential equation

$$\begin{array}{c} \dot{V}=\frac{1}{\tau }\left(\mu -V\right)+I_{syn} \end{array}$$ (1)

where $\dot{V}$ is the derivative of the membrane potential and *I*_*syn*_ is the total synaptic input to the neuron. The membrane time constant *τ* was set to 15 ms for excitatory neurons and 10 ms for inhibitory neurons. The bias *μ* was defined for each neuron by drawing from a uniform distribution with values between 1.1 and 1.2 for excitatory neurons and 1 and 1.05 for inhibitory neurons. These values helped ensure low mean firing rates similar to those observed in cortex [57]. A spike occurred when neurons reached the threshold *V*_*th*_ = 1, after which the neuron was reset to *V*_*re*_ = 0 with an absolute refractory period of 5 ms. Here we have normalized the voltages to range between 0 and 1, with a value of 0 corresponding to roughly -65 mV and a value of 1 corresponding to roughly -50 mV, as in biological networks.

For each neuron, total synaptic input current, $I_{i,syn}^{x}\left(t\right)$, to neuron *i* in population *x* at time *t* was defined as

$$\begin{array}{c} I_{i,syn}^{x}\left(t\right)=\sum_{jy}J_{ij}^{xy}F^{y}*s_{j}^{y}\left(t\right) \end{array}$$ (2)

where *x*, *y* ∈ {*E*, *I*} indicate populations of excitatory and inhibitory neurons and $J_{ij}^{xy}$ describes the synaptic weight from neuron *j* in population *y* to neuron *i* in population *x*. The convolution of the spike train, $s_{j}^{y}\left(t\right)$, with a filter, *F*^*y*^, is denoted by *. The filter, *F*^*y*^ is described by the equation

$$\begin{array}{c} F^{y}\left(t\right)=\frac{1}{\tau_{2}-\tau_{1}}\left(e^{-t/\tau_{2}}-e^{-t/\tau_{1}}\right) \end{array}$$ (3)

where time constants *τ*_2_ = 3 ms for excitatory synapses and 2 ms for inhibitory synapses and *τ*_1_ = 1 ms for all synapses, consistent with fast glutamatergic and GABAergic synaptic transmission. These values were selected to reproduce the effects of fast-acting excitatory and inhibitory neurotransmitters. One trial was defined as one second of time according to the simulation.

We simulated two network structures, one with homogeneous connection probability across excitatory neurons (non-clustered network) and one with clusters of high within-cluster connection probability (clustered network). In the non-clustered network, synaptic strengths were *J*^*EE*^ = 0.024, *J*^*EI*^ = −0.045, *J*^*IE*^ = 0.014, and *J*^*II*^ = −0.057. The probability of synaptic connection between excitatory neurons projecting onto other excitatory neurons occurred with probability *p*^*EE*^ = 0.2. All other types of synaptic connections occurred with probability *p*^*EI*^ = *p*^*IE*^ = *p*^*II*^ = 0.5. These connection probabilities are similar to the connection probabilities seen in cortex [58, 59]. When no synaptic connection existed between neurons, $J_{ij}^{xy}$ was set to zero.

In the clustered network, the probability of connection between excitatory neurons depended on whether two neurons were in the same cluster or in different clusters, with $p_{IN}^{EE}=0.4854$ for pairs within the same cluster and $p_{OUT}^{EE}=0.1942$ for pairs in different clusters (mean connection probability was *p*^*EE*^ = 0.2). This ratio of $p_{IN}^{EE}$ to $p_{out}^{EE}$ has been shown to maximize spiking variability for this network size [17]. Synaptic strength between excitatory neurons was $J_{IN}^{EE}=0.0456$ for within-cluster synapses and $J_{OUT}^{EE}=0.024$ for between-cluster synapses. These parameters were used ensure that cluster transitions led to spike train autocorrelation functions with decay timescales consistent with *in vivo* recordings [35]. All other synapses were set as specified above for the non-clustered network. The clustered network contained 50 clusters of 80 neurons each.

### Factor analysis

We used factor analysis (FA) to characterize the population activity structure [2, 4, 20–22]. In contrast to principal component analysis (PCA), FA explicitly partitions the spike count covariance into a component that is shared across neurons in the recorded population and a component that is independent across neurons [20]. This allows us to characterize the shared population activity structure (i.e., the shared component), which can be masked by Poisson-like spiking variability (i.e., the independent component) during single-trial activity. As a result, FA is more appropriate than PCA for analyzing single-trial spike counts [23].

FA is defined by:

$$\begin{array}{c} x∼N(\mu ,LL^{T}+\Psi ) \end{array}$$ (4)

where $x\in R^{n\times 1}$ is a vector of spike counts across the *n* simultaneously-recorded neurons, $\mu \in R^{n\times 1}$ is a vector of mean spike counts, $L\in R^{n\times m}$ is the loading matrix relating *m* latent variables to the neural activity, and $\Psi \in R^{n\times n}$ is a diagonal matrix of independent variances for each neuron. The model parameters ***μ***, *L*, and *Ψ* were estimated using the expectation-maximization (EM) algorithm [60].

As shown in Fig 1A, FA separates the spike count covariance into a shared component, *LL*^*T*^, and an independent component, *Ψ*. The rank, *m*, of *LL*^*T*^ indicates the number of latent variables needed to describe the shared covariance. If *m* equals one, then the shared covariance exists on a line. If *m* equals two, then the shared covariance exists on a plane, and so on. To determine *m*, we applied FA to the spike counts and selected the value for *m* that maximized the cross-validated data-likelihood.

In this study, we used two key metrics derived from the shared covariance matrix to describe population activity: shared dimensionality (*d*_*shared*_) and percent shared variance. First, we sought to measure the number of dimensions in the shared covariance as a metric for the complexity of the population activity. We followed a two step procedure to obtain this metric. We first found the *m* that maximized the cross-validated data likelihood, as is standard practice. We then defined *d*_*shared*_ as the number of dimensions that were needed to explain 95% of the shared covariance, *LL*^*T*^. We did this for the following reasons. In simulations, we found that, when training data were abundant, there was not a strong effect of overfitting and the cross-validated data likelihood curve saturated at large dimensionalities. As a result, the peak data-likelihood appeared at widely varying dimensionalities along the flat portion of the curve, leading to variability in the value of *m* from one run to the next. In contrast, we found that defining *d*_*shared*_ as described above provided a more reliable estimate of dimensionality across analyses, even if it may have been slightly smaller than the true dimensionality.

Second, we measured the amount of each neuron’s variance that was shared with at least one other neuron in the recorded population (Fig 1C). Mathematically, percent shared variance for the *k*^*th*^ neuron was computed as:

$$\begin{array}{c} \text{Percent}\;\text{shared}\;\text{variance}\;\text{for}\;\text{neuron}\;k=\frac{L_{k}L_{k}^{T}}{L_{k}L_{k}^{T}+\Psi_{k}} \end{array}$$ (5)

where *L*_*k*_ is the *k*^*th*^ row of the factor loading matrix and Ψ_*k*_ is the independent variance for the *k*^*th*^ neuron. The values reported in this paper (see Figs 3, 5, 6 and 7B) represent averages over all neurons included in a given analysis.

For Figs 1B, 4A, 8C and 8D, we computed a separate metric, the percent of overall shared variance explained by each mode. This was used to quantify the relative dominance of each mode for explaining shared variability. The percent of shared variance explained by the *i*^*th*^ mode was computed as:

$$\begin{array}{c} \%\text{ of}\;\text{shared}\;\text{variance}\;\text{explained}\;\text{by}\;\text{mode}\;i=\frac{\lambda_{i}}{\sum_{j=1}^{m}\lambda_{j}} \end{array}$$ (6)

where *λ*_*i*_ is the eigenvalue of *LL*^*T*^ corresponding to the *i*^*th*^ mode and *m* is the rank of *L*. Note that this metric does not take into account the independent variances. Throughout this work, modes are referred to as “dominant” if they explain a large percentage of shared variance.

In Figs 4C, 9C and 9D, we partitioned the percent shared variance (Eq 5) along each mode. We computed percent shared variance along the *i*^*th*^ mode for the *k*^*th*^ neuron as:

$$\begin{array}{c} \%\text{ shared}\;\text{variance}\;\text{for}\;\text{mode}\;i\;\text{and}\;\text{neuron}\;k=\frac{\lambda_{i}u_{ik}^{2}}{L_{k}L_{k}^{T}+\Psi_{k}} \end{array}$$ (7)

where *u*_*ik*_ is the *k*^*th*^ entry in the *i*^*th*^ eigenvector of *LL*^*T*^. We then averaged this value across all neurons. This allowed us to break down the contribution of each mode to percent shared variance and illustrated a contrast between the clustered and non-clustered network models. Note that Eq 5 is equivalent to summing Eq 7 over all *i*.

### Varying neuron and trial count

A central goal of this study was to determine how *d*_*shared*_ and percent shared variance vary with neuron and trial counts for *in vivo* recordings and spiking network models. To study these trends with changes in neuron count, we sampled increasing numbers of neurons from either the V1 recordings or the network simulations. FA was then applied to the selected neurons to obtain *d*_*shared*_ and percent shared variance. To increase the neuron counts, we augmented the next smaller sample of neurons with additional randomly selected neurons. For example, we first randomly selected 10 neurons, computed *d*_*shared*_ and percent shared variance for this set, and then added 10 additional randomly-selected neurons to obtain a new sample of neurons. For the *in vivo* recordings we repeated this procedure for each of three arrays, using 1200 trials for each neuron count.

For the model networks, we repeated this procedure 25 times at each neuron count using 5 non-overlapping sets of neurons and 5 non-overlapping sets of trials, using either 1200 or 10,000 trials for each neuron count. All neuron samples were obtained exclusively from the excitatory populations in the two networks. Since inhibitory neurons in both model networks did not have clustering structure, exclusion of this population allowed us to isolate the impact of clustering on the observed trends.

We studied how *d*_*shared*_ and percent shared variance change with trial count by performing the same procedure as described above, except that trials were increased rather than neurons. For *in vivo* recordings, we repeated this procedure once for each of three arrays with the same 80 neuron sampled in all trial counts. In the model networks 25 analyses were run with 5 non-overlapping sets of 80 neurons and 5 non-overlapping sets of trials for each trial count.

### Modes of shared population activity structure

We sought to assess how the identified modes of shared variance change with increasing neuron count. We could do this by measuring the angles between corresponding modes; however as neuron count increases, modes can change order, causing direct angle measurements between modes with the same index to overestimate the difference between the two sets of modes.

To overcome this, we measured principal angles between sets of modes [61]. We first identified the modes of shared variability as the eigenvectors of the shared covariance matrix *LL*^*T*^. We then computed the principal angles between modes from two different conditions (i.e., different numbers of neurons). Since the vector defining each mode had length equal to the number of neurons in the sample, we could not directly measure the principal angle between the eigenvectors of conditions with different neuron counts. To overcome this, we first computed *LL*^*T*^ in each condition using only the rows of *L* that correspond to a set of 20 neurons common across the 20-, 40-, 80-, and 500-neuron sets studied. Once we computed the reduced *LL*^*T*^ in each condition, we then measured the angle between the modes as described above. Additionally, to restrict the analysis to the most dominant modes, only the five modes explaining the largest amount of shared variance were included in all principal angle measurements.

### Distribution of neurons across the network

To study how the choice of sampled neurons influences *d*_*shared*_ and percent shared variance, we sampled excitatory neurons from the clustered network by varying the number of clusters represented in a set of 50 neurons sampled. This was done by first randomly selecting *N* clusters from the total of 50 clusters in the network. Next, 50/*N* excitatory neurons were selected at random from each of the chosen clusters. The variable *N* was selected so that 50/*N* resulted in an integer value. FA was applied to 10,000 trials from the 50 selected neurons. The procedure was repeated 25 times with 5 non-overlapping sets of neurons and 5 non-overlapping sets of trials.

## Supporting Information

S1 Fig Trends in *d*_*shared*_ and percent shared variance are robust to time bin size. To assess the effects of spike count bin size on the observed results, we repeated the analyses in Fig 3 for 200 ms and 500 ms bins. We measured *d*_*shared*_ and percent shared variance over a range of (A) neuron counts and (B) trial counts from population activity recorded in V1 with spike counts taken in 1000 ms (black), 500 ms (blue), and 200 ms (red) bins. Each triangle represents the mean across single samples from each of three arrays. Error bars represent one standard error across the three arrays. We observed trends of increasing *d*_*shared*_ and stable percent shared variance with neuron and trial counts for all bin sizes, implying that the observed trends are consistent across a range of timescales. Furthermore we found that *d*_*shared*_ and percent shared variance were lower for smaller bin sizes, consistent with previous work showing that noise correlations scale with bin size [26, 38].
(EPS)

S2 Fig Sequential and non-sequential time bin samplings yield similar *d*_*shared*_ and percent shared variance. Factor analysis does not take into account sequential relationships between time bins. In other words, it assumes that the spike counts in consecutive time bins are independent of one another. We found that this assumption is valid for 1-second time bins using the following analyses. (A) We computed the autocorrelation of the spike counts in 1-second bins and found near-zero auto-correlation at all non-zero lags. Black line represents mean across 80 neurons from 3 arrays (*n* = 240) and vertical bars represent one standard error across all neurons from three arrays (*n* = 240). In addition, we replicated the analysis in Fig 3B corresponding to 400 trials (mean *d*_*shared*_ of 4.3, mean percent shared variance of 51.8%, labeled here as ‘Random’) using three different trial sampling methods: (1) sampling of adjacent trials (‘Skip 0s’), (2) sampling trials separated by 1 second (‘Skip 1s’), and (3) sampling trials separated by 2 seconds (‘Skip 2s’). We used 400 trials in each case. Triangles represent mean across 3 arrays and error bars represent one standard error across the three arrays. All of these measures produced qualitatively similar results across these sampling methods and no sampling method was significantly different from the random set (*p* > 0.05 in all cases). Taken together, these results suggest that the 1-second bin size is large enough such that successive bins are effectively independent, and our use of random time bins did not impact our results.
(EPS)

S3 Fig *In vivo* recordings do not show clear cluster structure. (A) We applied the k-means algorithm to the rows of the modes matrix in Fig 4A in an attempt to identify clusters of neurons in the *in vivo* recordings. Rows are sorted according to groups identified by the k-means algorithm for *k* = 5. (B) Same convention as in (A) except *k* = 10. (C) To assess whether there is clustering among neurons in the *in vivo* recordings, we applied linear discriminant analysis to find the 2-dimensional projection of the 10-dimensional row vectors with the best separability of the five groups identified by k-means in (A). Each circle corresponds to a neuron, and each color corresponds to a group identified by the k-means algorithm. Although neurons assigned to the same group tend to have similar 10-dimensional vectors, we saw no clear separation of the groups identified by k-means. Repeated random initialization of k-means yielded different groupings among the neurons, further indicating that there is no clear clustering among the neurons. Note that the fact that points with the same color lie near each other in the 2-dimensional projection is not an indication that there is clustering among neurons. This would result from applying the k-means algorithm to any scatter of points. The key is whether the groups identified by k-means are well-separated. (D) Same analysis as in C for the groups shown in (B).
(EPS)

S4 Fig Modes of shared variability for clustered network model in experimental regime of neuron and trial counts. (A) Principal angles between top five modes in clustered network for 20- (blue), 40- (black), or 60-neuron (red) analysis and corresponding neurons from 80-neuron analysis. Modes were identified by computing the eigenvectors of the shared covariances corresponding to neurons from the 20-neuron set. Plots show mean and standard error across 25 sets of 80 neurons and 1200 trials. Grey circles represent principal angles (mean ± one standard deviation) between random 20-dimensional vectors. (B) Percent shared variance along each mode in the clustered network for the 20-neuron analyses (blue) and the 80-neuron analyses (black) shown in (A).
(EPS)
